# Identifying frailty in primary care: a qualitative description of family physicians’ gestalt impressions of their older adult patients

**DOI:** 10.1186/s12875-018-0743-4

**Published:** 2018-05-14

**Authors:** Clara Korenvain, Ida-Maisie Famiyeh, Sheila Dunn, Cynthia R. Whitehead, Paula A. Rochon, Lisa M. McCarthy

**Affiliations:** 1 Women’s College Hospital, 76 Grenville Street, Toronto, ON Canada; 2 Leslie Dan Faculty of Pharmacy, University of Toronto, 144 College Street, Toronto, ON Canada; 3 Faculty of Medicine, University of Toronto, 1 King’s College Circle, Toronto, ON Canada; 4 Women’s College Research Institute, 76 Grenville St, Toronto, ON M5S 1B2 Canada; 5 The Wilson Centre, University Health Network, 200 Elizabeth Street, Toronto, ON Canada

**Keywords:** Frailty, Qualitative, Primary care, Family physicians, Older adults

## Abstract

**Background:**

Many tools exist to guide family physicians’ impressions about frailty status of older adults, but no single tool, instrument, or set of criteria has emerged as most useful. The role of physicians’ subjective impressions in frailty decisions has not been studied. This study explores how family physicians conceptualize frailty, and the factors that they consider when making subjective decisions about patients’ frailty statuses.

**Methods:**

Descriptive qualitative study of family physicians who practice in a large urban academic family medicine center as they participated in one-on-one “think-aloud” interviews about the frailty status of their patients aged 80 years and over. Of 23 eligible family physicians, 18 shared their impressions about the frailty status of their older adult patients and the factors influencing their decisions. Interviews were audio-recorded, transcribed, and thematically analyzed.

**Results:**

Four themes were identified, the first of which described how physicians conceptualized frailty as a spectrum and dynamic in nature, but also struggled to conceptualize it without a formal definition in place. The remaining three themes described factors considered before determining patients’ frailty statuses: physical characteristics (age, weight, medical conditions), functional characteristics (physical, cognitive, social) and living conditions (level of independence, availability of supports, physical environment).

**Conclusions:**

Family physicians viewed frailty as multifactorial, dynamic, and inclusive of functional and environmental factors. This conceptualization can be useful to make comprehensive and flexible evaluations of frailty status in conjunction with more objective frailty tools.

**Electronic supplementary material:**

The online version of this article (10.1186/s12875-018-0743-4) contains supplementary material, which is available to authorized users.

## Background

Clinical gestalt, also referred to as a “gut feeling” or “intuition,” is a reasoning method whereby healthcare practitioners actively organize clinical perceptions into a coherent whole [1]. It is a form of heuristic pattern recognition that allows efficient decision-making even with limited information [1, 2]. Gut impressions have been subdivided into feelings of alarm, which can prompt further investigation and action on the part of the provider, and reassurance in the face of an uncertain prognosis or diagnosis [2, 3]. Its usefulness has been compared to common decision rules for identifying patients with venous thromboemboli [4–6]. Some argue that gestalt can lead to systematic errors in judgment and advocate to minimize clinician reliance on this method, while others assert that it is a necessary and useful part of clinical practice [1, 2]. Enhancing understanding about gestalt in the context of frailty is important because for most family practices which lack a structured process for identifying frailty, clinicians are using gestalt to identify frailty among older adults.

While frailty is widely recognized as a clinical syndrome, there is no consensus on an operational definition, nor a gold standard tool to identify or measure it. Frailty is gaining increasing attention internationally due to its association with adverse health-related outcomes [7] including functional decline, dependency, recurrent falls, fractures, poor outcomes after discharge from emergency rooms, recurrent hospitalizations, institutionalization, and death [8, 9]. It is estimated that globally, approximately 10% of community-dwelling adults aged 65 and over are frail [10].

Developing a pragmatic approach for identifying frailty within the existing constraints of primary care settings is important because family physicians are the main providers of comprehensive healthcare to increasing numbers of older adults. Multiple tools evaluating frailty have been tested in a variety of settings. They differ widely in their features (e.g. length, time to complete, phone or in-person mode of administration), domains included, and outcomes measured. No one tool has emerged as optimal for identifying frailty in primary care and it is not clear if any of the instruments will perform better than physicians’ judgment.

In the context of screening for frailty, some studies suggest that clinical gestalt can be useful for predicting poor health outcomes [11, 12]. However, physicians’ gestalt has not been described beyond a categorical decision with respect to a patient’s frailty, or without compromising the integrity of gestalt by guiding physician notions of frailty through the research process. Understanding the themes that arise when family physicians apply gestalt to their older adults is helpful for understanding how physicians’ conceptualization of frailty is similar or different from existing frailty tools. Further, this understanding contributes to discussions about the importance of investment in screening strategies that are more costly and less easily implemented within practice.

This paper reports the qualitative component of a multi-methods study called INFER-PC (**I**de**n**tifying **F**railty Amongst Old**er** Adults in **P**rimary **C**are), conducted between November 2014 and December 2015, which aimed to: 1) compare the usefulness of existing frailty measures to identify an appropriate screening tool for use with older adults in primary care settings, and 2) assess the relationship between family physicians’ clinical impressions of frailty relative to classifications from the existing frailty measures, 3) describe factors influencing family physicians’ impressions of frailty in their older adult patients. In this report, we describe themes that emerged when family physicians were asked to share their gestalt impressions about the frailty status of their patients aged 80 and over. Specifically, we were interested in how family physicians conceptualize frailty, as well as what factors they consider when making decisions about frailty status.

## Methods

The study was conducted within a large family medicine clinical teaching unit that has served its more than 20,000 patients in Toronto, Canada for over 20 years. Patients are assigned to a most responsible physician but have access to many onsite interprofessional care providers including nurses, dietitians, social workers, a pharmacist and an occupational therapist. All permanent staff physicians who were registered for a minimum of 6 months as the most responsible care provider for at least one community-dwelling adult aged 80 and over were invited to participate via email. For the purposes of the study, community dwelling adults were those who lived alone, with a caregiver, or with others (including patients receiving home care services).

A qualitative research design was used for study. Analysis of linguistic data provides non-numerical answers to research questions such as *how* physicians conceptualize frailty and *what* factors they consider when assessing frailty status [13]. Since there are no previous studies exploring physicians’ gestalt about frailty, a broad exploratory approach was undertaken for this study using a low-inference descriptive qualitative methodology [14].

Consenting physicians participated in individual face-to-face sessions at their workplace lasting approximately 30 min. A non-clinician research assistant with experience interviewing health care professionals and familiarity with the medical team through other research projects conducted the sessions. During the sessions, physicians were asked to review and identify from their list of patients (generated from the electronic medical record) those who satisfied (or did not satisfy) the patient inclusion criteria (i.e., who was frail). To explore clinical gestalt, a think aloud approach was used to gather physicians’ views about the frailty statuses of their patients. Think-aloud is “a research method in which participants speak aloud any words in their mind as they complete a task” [15]. The process involves working from memory, which has been shown to be the most effective way to assess higher-level thinking processes [15]. In this study, physicians were given the following prompt (developed by a multidisciplinary group of research team members including a pharmacist and several physicians, see Additional file 1 Interview Guide) and asked to respond, “yes”, “no”, “maybe” or “I do not know”: “*There are many definitions of frailty, and many factors to consider in categorizing someone as vulnerable or frail. Using your clinical judgment, which of these older patients do you consider to be frail or at-risk for frailty?”* For patients considered frail, physicians were asked to share the reason(s) if known. To maintain the integrity of the think-aloud method, physicians were not probed further after the initial prompt.

Responses to clinical gestalt questions were compiled and themes developed using a thematic analysis approach [16, 17]. Interviews with physicians were audiotaped and transcribed verbatim, de-identified, then coded inductively using conventional word processing software [17]. To develop the initial coding scheme, including broad themes and subthemes, one senior research team member (L. McCarthy, a pharmacist-researcher with experience in qualitative research) and a pharmacist/Master of Science student (I. Famiyeh – studying qualitative research) reviewed the first 10 transcripts. IF used this scheme to code the remaining transcripts. A third team member (C. Korenvain, pharmacist and Master of Science student studying qualitative research) independently reviewed all of the transcripts and participated in revising the scheme with I. Famiyeh as the analysis progressed and new themes emerged. All researchers conducting and analyzing the interviews were female.

Sample size was determined by the number of physicians who agreed to be interviewed, out of 23 that were eligible. The researchers felt that saturation of themes was achieved after analyzing all the interviews, especially since the sample was not diverse with respect to context (from a single family practice clinic) [18].

The study was approved by the Women’s College Hospital Institutional Research Ethics Board, 2013-0015-B.

## Results

Of 23 eligible physicians, 18 agreed to participate and were interviewed. Fourteen were female and the median number of years of experience as a family physician was 15 (minimum 3 years; maximum 41 years).

**Fig. 1:**
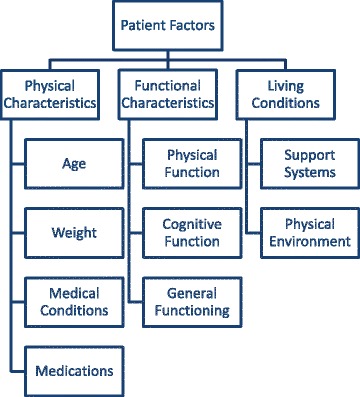
Factors considered by family physicians when making decisions about frailty status

Emergent codes were grouped into four descriptive themes. One theme, physicians’ concept of frailty (spectrum, dynamic, uncertainty), described how physicians conceptualized or defined frailty. The remaining three themes described factors that physicians considered before determining patients’ frailty statuses (Fig. 1). These patient factors include: physical characteristics (age, weight, medical conditions, and medications), functional characteristics (physical, cognitive, general) and living conditions (availability of needed support systems, nature of physical environment).

### Physicians’ concept of frailty

Three subthemes were produced from physicians sharing their thoughts about what frailty means to them: spectrum, dynamic, and uncertainty.

*Spectrum:* This subtheme describes physicians’ concept of frailty as being on a continuum or having different levels. One physician stated, “She’s got a fair bit going on, again, there is a continuum” (Physician 3). Other physicians used unique terms to refer to different levels of frailty, such as “frailish” (Physician 4), “somewhat frail” (Physician 15), “at risk of frailty” (Physician 12), and “minimally frail” (Physician 16).

*Dynamic:* Physicians also described frailty in terms of how it changes in status over time. One physician talked about worsening frailty: “she […] had a rapid decline recently and is now in palliative care home and probably going into the hospice in the next few days” (Physician 3). Sudden changes in status could occur after a single life event such as a myocardial infarction or sudden vision loss. While some physicians talked about improvement in frailty, others suggested that frailty could not always be improved: “It’s not the kind of frailty that we improve on; he’s getting worse no matter what...” (Physician 9).

*Uncertainties:* This subtheme refers to when physicians had uncertainties about the definitions of frailty or could not make a decision. One physician acknowledged the uncertainty and lack of reliability in making a decision about a patient’s frailty status: “I mean it would be interesting if you guys [researchers] meet these people and have a completely different perception than I do because maybe I’m wrong you know” (Physician 15). Another physician described improvement in frailty contrary to expectations:

“I think she may be [frail], but she’s working on it...she couldn’t get out of her chair very well and so I asked her that every time she went to the toilet to practice standing up and sitting down five times and she came back 2 weeks later and she said “It’s a miracle” and she can actually, she’s much better now just because I got her to do a very simple exercise” (Physician 9).

Another source of uncertainty was around how much emphasis should be given to one factor versus another, “…frailty, how much is it a cognitive thing, how much is it a physical thing?” (Physician 3). Although Physician 3 considered both cognitive and physical aspects of frailty, other physicians defined it as purely a physical entity, “frailty is a physical thing… I mean I thought frailty might encompass the cognitive but in my conception of frailty it’s a physical issue not a dimension in cognition” (Physician 8). There were times when physicians would consider patients as both frail and not frail. “…I would say no because she’s independent but yes because she’s totally blind and can’t do some things” (Physician 17). One physician correlated frailty with patients’ visit frequency, “I think people that haven’t come in for a long time probably are frail and that’s why they haven’t come in…Because they are not easily able to come in cause the less frail seniors I see very often; more for social stimulation and visits, whereas the more frail find it harder to come in” (Physician 19).

### Factors considered in frailty decision-making

#### Physical characteristics

This theme describes physical characteristics that physicians took into consideration while deciding on their patients’ frailty statuses.

*Age:* Most physicians considered their patients’ age in their frailty decisions. While most physicians considered age as a risk factor for frailty among other factors, “…he’s ninety-two and he’s got cardiovascular disease, congestive heart failure, degenerative disk disease of his lower back” (Physician 6), others based their decisions mainly on their patients’ older age: “I would say yes...more because of age...events due to her age and her status” (Physician 11). “She must be what like ninety-two now or something? She is getting up there...Yeah I would qualify her as frail” (Physician 15). In contrast, some physicians did not consider their patients as frail despite their older age. One physician stated, “I don’t think he’s frail even though he’s eighty-four” (Physician 1).

*Weight:* Seven physicians factored in their patients’ weight in their thought processes and decisions about frailty. Five physicians placed emphasis on weight and considered patients who are “thin” as being frail. One physician mentioned, “if they’re thin I think they’re frail...I have a hard time thinking about someone who is big as being frail and I might classify someone who’s thin with the same level of function as being frail” (Physician 8). For other physicians, a patient’s weight was not the overriding factor, “she is incredibly frail.... she’s huge but frail” (Physician 4). Likewise, another physician expressed: “...is she frail? I’m going to say no, she has COPD [Chronic Obstructive Pulmonary Disease], she’s very thin, but cognitively fully intact...functions well, has good social supports” (Physician 10).

*Medical Conditions:* Patients’ medical conditions influenced the thought processes of 15 physicians. Some physicians considered the presence of multiple medical conditions as a contributing factor to frailty: “...has multiple chronic things but still manages to function quite well but she’s certainly at risk” (Physician 3). Others made a decision based on the consequences of a single medical condition: “she is incredibly frail...she has the worst gout I’ve ever seen and she’s very disabled” (Physician 4). The presence of a specific medical condition did not always lead to the conclusion that a patient was frail, “I think...is not frail although she has had cancer...” (Physician 4).

*Medications:* Only one physician considered patients’ number of medications in deciding about frailty: “...is frail for sure, so she’s post stroke, um has many meds, cognition is okay but...her ability to function independently is probably limited but she’s well supported” (Physician 10).

#### Functional characteristics

Subthemes about patients’ functional characteristics that emerged during the physician interviews were physical, cognitive, and general function.

*Physical Function:* This subtheme refers to situations in which physicians considered their patients’ ability to carry out physical tasks. Physical function was mainly discussed in terms of mobility. While some patients were considered to be frail due to issues with mobility, “...yes, limited ambulation and has help with all her IADLs [Instrumental Activities of Daily Living]” (Physician 2), others were not: “...I don’t know if I would define her as frail, she does have mobility issues, she is a falls, a big falls risk but she lives on her own, she is fiercely independent...” (Physician 1).

*Cognitive Function:* Twelve physicians talked about their patients’ cognitive function. For some physicians, the presence of cognitive impairment alone puts a patient at risk of frailty, “she still goes and exercises...but she’s had some cognitive issues, mild cognitive stuff.... the cognitive stuff is a risk, the physical stuff she’s been amazing” (Physician 3). Others listed cognition among other factors that contribute to frailty: “…is frail, so he’s got mobility, cognition…he’s fallen many times, and he’s got, so diabetes, seizures, so like multiple conditions, poly-pharmacy, lots of side effects from drugs” (Physician 10).

*General Function*: This subtheme describes discussions about function that did not specifically fall under physical or cognitive function. Physicians often talked about function in terms of level of independence. Most physicians concluded that their patients were not frail because they functioned well independently, “she’s actually quite independent, quite high functioning, she has a chronic underlying condition and she has arthritis, so she has some limitations from arthritic knees but she’s pretty high functioning and so to me she is not particularly frail” (Physician 11). Similarly, the inability to function independently was the reason some patients were considered frail: “she’s reliant on her caregivers but yeah I think she was, she I would…Define her as frail” (Physician 1). For other physicians however, patients’ ability to function independently did not automatically make them non-frail: “This is a maybe, she’s a maybe, she’s in, in some ways she’s no because she functions independently, but in some ways she’s yes because she has multiple hospital admissions” (Physician 2).

#### Living conditions

In terms of living conditions, physicians considered patients’ availability of needed support systems and the nature of their physical environment.

*Availability of support systems:* This subtheme refers to instances when physicians considered the presence or absence of a patient support system in their decisions about frailty. The support systems were social (family members, friends) and professional (e.g., home care). The presence of a support system was seen as a sign of robustness protecting the patient against worsening frailty: “she is frail; she copes because her husband looks after her” (Physician 14). “[The patient] is frail for sure...but she has good family support. Just her ability to function independently is probably limited but she’s well supported” (Physician 10). Similarly, patients who lacked support systems were perceived to be frail, “like socially he is frail...like he just needs supports” (Physician 19). In contrast, for some physicians, the fact that a patient required support indicated his/her frailty status, “... is the frailest person I know, she has twenty-four hour care” (Physician 4).

*Physical Environment:* Two physicians took into consideration their patients’ physical environment during their thought processes about frailty. “[She] is definitely a yes, lives in a three story house, and is extraordinarily resistant to moving...she wouldn’t let me in when I went to see her because it is packed with papers I heard” (Physician 4).

## Discussion

### Summary

This study provides insight into the thought processes of family physicians that underlie their clinical impressions of the frailty status of their older patients. Physicians conceptualized frailty as on a spectrum and dynamic in nature, but also faced uncertainties in trying to identify frail patients or conceptualizing frailty. Physicians took a number of factors into consideration before deciding on whether or not their patients were frail, including physical characteristics (age, weight, medical conditions), functional characteristics (physical, cognitive, social) and living conditions (level of independence, availability of supports, physical environment).

### Strengths and limitations

To maintain the integrity of the think-aloud approach, physicians were not probed to provide in-depth explanation during the interviews. The absence of probing could have prevented automatic thinking patterns from being verbalized [15]. This led to some physicians listing different risk factors of frailty without discussing the extent to which the factors influenced their decisions. Despite being asked to explain the reasoning behind their decisions about frailty, some physicians may have considered additional factors that were not verbalized, and would therefore only provide partial insight into their thought process. It is possible that participants only articulated the most prominent factors in their decision-making process for a particular patient case.

The study findings do not necessarily apply to all family physicians. All interviewed physicians work in a single large urban family practice clinic, limiting generalizability of findings to different contexts. The physicians in this study are mostly experienced and seemed familiar with their patients’ health profiles. More experienced clinicians are more adept at pattern recognition within their area of expertise and may be more reliant on gestalt [1, 2]. Having immediate access to more information about patients through familiarity and experience with them may have increased their confidence in their decision-making about frailty.

The investigators analyzing study results (I. Famiyeh, C. Korenvain and L. McCarthy) have all practiced at the study site family practice clinic or in similar settings as pharmacists or pharmacy students, and may therefore have their own gestalt impressions of frailty. Although none of these researchers were familiar with the specific patients discussed or had close relationships with the physicians interviewed, their personal conceptualizations of frailty may have influenced the analysis. For example, CK admits that the physicians’ responses have expanded her conceptualization of frailty to include a patient’s environment. This finding was discovered in part because it surprised the researcher by contrasting with her own preconceived notions.

### Comparison with existing literature

Physicians’ conceptualization of frailty as being on a spectrum and dynamic in nature has been reported previously in the literature. As Rouge et al. state, “frailty should be considered as a dynamic and potentially reversible process and as a continuum with intermediate states that can be modified (robustness <-> frailty <-> disability)” [19]. Physicians’ clinical impressions can in this way introduce flexibility into frailty screening in primary care, which a one-time or scheduled periodic assessment via objective tools may not provide.

In general, physicians did not attribute one factor to be the sole determinant of their patients’ frailty statuses. They often considered other contributing factors and made a decision based on which factor they deemed most important for that patient. Risk factors for frailty that were given greater emphasis were those that appeared to affect patients’ ability to function in general. For example, medical conditions that were associated with frailty were those that were disabling (gout, osteoporosis). This is consistent with some literature definitions of frailty, which state that “frailty can be thought of as a combination of factors that influence a person’s physiologic state to the extent that function is greatly reduced and the person becomes more vulnerable to external stressors” [20].

Participants considered patient factors in their decisions that were beyond the scope of traditional definitions of frailty based solely on physiology [7, 21]: living conditions and functional characteristics. The incongruence between research and practitioner conceptualization of frailty has been described previously [21]. Some researchers have problematized the fact that existing frailty tools do not seem to distinguish between physiologic vulnerability and functional compromise, and do not always take into account factors outside the patient [21]. Subjective frailty determinations by US physicians highly correlated with a lack of social support noted in the patient’s EMR [Electronic Medical Record] [11], which is consistent with this study’s finding that physicians’ gestalt impressions of frailty encompass factors beyond physiology.

### Implications for research and practice

Physicians’ conceptualization of frailty as dynamic and on a spectrum introduces more flexibility into frailty assessment than existing objective tools can provide. Since some participants described changes in frailty status that occurred immediately following a significant medical or psychosocial life event, clinical gestalt could prompt a re-evaluation of frailty status and the need for additional supports in an ad hoc fashion. The long-term relationships that family physicians have with patients provide opportunities for monitoring frailty status over time, and clinical gestalt can facilitate this process.

Accounting for a broad range of patient and environmental factors, as family physicians’ gestalt did in this study, may prompt family physicians to link patients, who are functionally limited or living in suboptimal conditions, with supports to address these issues and thus reduce the risk of adverse health outcomes.

This study does not provide evidence to support or refute using clinical gestalt as a replacement for more objective frailty measures. Comparisons between family physicians’ gestalt decisions about frailty and the results of more objective frailty tools will be reported elsewhere. However, the dynamic conceptualization of frailty by physicians allowing assessment flexibility, as well as their attention to functional and environmental factors contributing to patient vulnerability, suggests that clinical gestalt is a valuable addition to a physicians’ frailty assessment toolkit.

## Conclusions

This study provides insight into the factors family physicians consider when making frailty determinations in the course of their daily practices, and how they conceptualize frailty. Findings contribute to a broader discussion about effective approaches to identify and address frailty in older patients by introducing how clinical gestalt can be used to compliment frailty identification and prediction by more objective tools. Study findings could contribute to the development of future models that would acknowledge, and perhaps make use of, clinical gestalt as part of a process used by family physicians to assess and monitor frailty in their patients, to optimally support their ongoing health and safety.

## Additional file

Additional file 1: Interview Guide. Question used for interviewing participants. (DOCX 12 kb)

## Abbreviations

COPD: Chronic obstructive pulmonary disease
EMR: Electronic medical record
IADL: Instrumental activities of daily living
INFER-PC: **I**de**n**tifying **F**railty Amongst Old**er** Adults in **P**rimary **C**are Study
