# Supplementary material for: Identifying frailty in primary care: a qualitative description of family physicians’ gestalt impressions of their older adult patients
Source: BMC Fam Pract. 2018 May 14;19:61. doi: 10.1186/s12875-018-0743-4 (PMC5952517; doi:10.1186/s12875-018-0743-4)
Supplement: Supplementary file 1 — Interview Guide. Question used for interviewing participants. (DOCX 12 kb) [file 12875_2018_743_MOESM1_ESM.docx]

**Interview Guide**

There are many definitions of frailty, and many factors to consider in categorizing someone as vulnerable or frail. Using your clinical judgment, which of these older patients do you consider to be frail or at-risk for frailty? Please answer “yes”, “no”, “maybe”, or “I do not know” for each patient.

For patients considered frail, please ask physicians to share the reason(s) if known.
